# S100 A and B expression in normal and inflamed human limbus

**Published:** 2013-01-28

**Authors:** Mario Nubile, Manuela Lanzini, Roberta Calienno, Rodolfo Mastropasqua, Claudia Curcio, Alessandra Mastropasqua, Luca Agnifili, Leonardo Mastropasqua

**Affiliations:** 1 Department of Medicine and Science of Ageing, Ophthalmology Clinic, University “G. D'Annunzio” Chieti-Pescara, Italy; 2 Ophthalmology Clinic, University of Verona; 3 Ophthalmology Clinic, Campus Biomedico, Roma

## Abstract

**Purpose:**

To study the expression of S100 A and B family proteins in normal human limbus and to analyze modification of the expression in inflammatory conditions.

**Methods:**

The total expression of members of the S100 family and the expression of A4, A8, A9, and B individually were evaluated in nine normal human corneal limbi, collected from cadaver healthy donors, in particular in the limbal epithelial crypts (LECs), and in five inflamed limbi obtained from enucleated eyes. S100 protein distribution was determined with immunohistochemistry staining analysis.

**Results:**

Cytoplasmic expression of total S100 proteins was observed in 100% of LECs; in contrast, the inflamed tissues were completely negative, and faint positivity was observed in only one case. Moreover, cytoplasmic expression of S100 A4 and A9 was uniformly found in the entire LECs in all samples analyzed, while S100 A8 positivity was observed in only 44.4% of cases and only in the cells localized in the central area of the LEC. Positivity for S100 B was not observed in all samples analyzed.

**Conclusions:**

As reported in the literature, normal limbal epithelial cells show strong expression of S100 proteins. A novel finding of this study was the expression for the limbal epithelial crypts. In particular, S100 A4 and A9, which are normally involved in regulating a wide range of biologic effects, including cell motility, survival, and differentiation, are the most expressed members in healthy limbal crypts. In inflamed tissues, expression of S100 proteins was dramatically decreased. S100 proteins, and in particular S100 A4 and S100 A9, can be useful as markers of early changes in stem cell niches due to inflammation.

## Introduction

Calcium ions act as ubiquitous second messengers in cells and trigger a multitude of cellular processes including secretion, contraction, metabolism, cell division, and cell growth. Calcium binding proteins are intracellular receptor molecules and couple changes in intracellular calcium levels with alterations in cell functions [1].

The S100 protein family represents one of the largest subfamilies of the EF-hand calcium-binding proteins, approximately 10,000 Da in size, with at least 19 different members with 50% homology in amino acid sequences [2]. Each member of the family exhibits a unique pattern of expression with some cells expressing multiple members of the family [1]. S100 proteins interact with other proteins to modulate various biologic functions and are thus related to various diseases, many of which involve inflammation, innate immunity, tissue damage, wound healing, stress response, cell motility, proliferation, and differentiation [3].

Although numerous studies have reported the physical properties, functions, and expression of some of these proteins (for example, S100 A1, S100 A8, S100 A9, and S100 B), for others little is known (for example, S100 A3 and S100 A5) [1]. Increased expression of different S100 proteins was identified in many cancers. For example, increased levels of S100 proteins were found in melanomas and acute leukemic change of myelocytes, increased levels of S100 B were reported in thyroid carcinoma, and increased levels of S100 A1 were reported in renal carcinoma [4]. Furthermore, many studies have reported that S100 proteins are implicated in regulating tumor cell proliferation and metastasis and are indirectly related to the prognosis of the disease; the lower the concentration of S100, the longer patients survive [1].

An increased level in S100 protein expression has also been found in different parts of the ocular system [5]. Several studies reported the expression of S100 proteins on the ocular surface in pathological inflammatory and non-inflammatory conditions. For example, increased expression of S100 A2 [6] and A4 has been found in the keratoconus corneal epithelium [7]. S100 A4, S100 A6, S100 A8, S100 A9, and S100 A13 were upregulated in corneal neovascularization murine models [8]. S100 A4 was reported to be overexpressed in keratocyte phenotypes that appear in stromal tissue of corneas recovering from damage, and it was hypothesized that S100 A4 is involved in the corneal wound healing process [9].

An increased level of S100 A6, A8, and A9 has also been identified in pterygium [10] compared with normal conjunctiva and of S100 A8 and A9 in tear samples of patients with pterygium compared to controls [11]. An abnormal level of S100 has also been detected in some rare ocular surface tumors [12] in the tears of patients with dry eye [13] and in the tear film of contact lens wearers (in particular S100 A8) [14]. Thus, numerous studies support the hypothesis of the involvement of S100 A proteins in inflammatory processes of the ocular surface.

The corneal epithelium is continuously renewed by a pool of cells with proliferative potential that reside in the basal epithelial layer of the human corneoscleral limbus in specific anatomic structures called limbal epithelial crypts (LECs) [15]. A recent study by Jing and colleagues reported the expression of S100 A proteins in normal corneal limbal epithelial cells and found it to be similar to that in normal skin [16].

The goal of our study was to investigate the presence of the S100 protein family and to quantify the levels of A4, A8, A9, and B in normal human LECs. We also evaluated modification of the expression of these proteins in inflammatory conditions.

## Methods

### Healthy and pathological samples collection

This study adhered to the tenets of the Declaration of Helsinki. The protocol was approved by our local ethical committee. Nine human eye bank corneal buttons with scleral rims, not suitable for transplantation (mean age 69.6±9.8 years, ages ranged from 52 to 80), were included in the study. The average death to enucleation time was 8 h (range from 4 h to 10 h). The mean storage (Eusol-C, Alchimia Srl, Ponte San Nicolò, Italy) time (between eye bank procedures and fixation) was 26 h (range 20 h to 48 h). No evidence of disease, desiccation, or damage was noted. All tissue was fixed in 4% formalin (Bio Optica, Milan, Italy).

In addition, five limbus samples were collected from pathological corneosclera (mean age 69.4±9.6 years, age ranged from 59 to 85) at the time of the surgical procedures of enucleation of the eye due to uncontrolled infectious endophthalmitis affecting the cornea and the ocular surface. Tissues were fixed in formalin. The etiology of endophthalmitis was originally related to corneal infection and included *Stenotrophomonas maltophilia*, *Aspergillus flavus*, *Candida albicans*, *Pseudomonas aeruginosa*, and one case of Herpes simplex virus necrotizing keratitis with cornea perforation and subsequent microbial unidentified superinfection. All pathological eyes presented variable degrees of limbal inflammation. Inflammatory cell phenotypes were determined by the presence of macrophages, granulocytes, and lymphocytes, as assessed with immunohistochemistry staining (data not shown).

Healthy donors were serially cut with a microtome (Leica Microsystems GmbH, Wetzlar, Germany), and monitored for the presence of LEC by staining of every 10th to 15th section with hematoxylin and eosin. When a region containing the LEC was identified, the adjacent sections were collected for immunohistochemistry staining. In addition, a p63 stain was performed to confirm the presence of LEC (supplementary data).

### Histological staining analysis

Immunohistochemistry analysis was performed on sections in which we observed the presence of LECs with hematoxylin and eosin staining and for all inflamed samples. Sections were stained in triplicate for the following antibodies: S100 (1:400, Dako, Glostrup, Denmark), S100 A4 (1:400, Abcam, Cambridge, England), S100 A8 (1:500, Sigma-Aldrich, St. Louis, MO), S100 A9 (1:100, Abcam), and S100 B (1:300, Abcam). Formalin-fixed tissues were deparaffinized and pretreated with microwave antigen retrieval using sodium citrate buffer for pH 6 (for S100) or EDTA for pH 9 (required for S100 A4, S100 A8, and S100 B). For all these antigens, the EnVision Rabbit system (Dako) was used before diaminobenzidine tetrahydrochloride (Dako) incubation. A negative control was performed for each antigen using the specific isotype control. All slides for the same antigen were stained together, with the same required antigen retrieval buffer and antibody dilution. The slides were examined in a double-blind fashion, and digital images of representative areas were taken.

## Results

### Expression of total S100 proteins in healthy and inflamed human limbus

**Figure 1:**
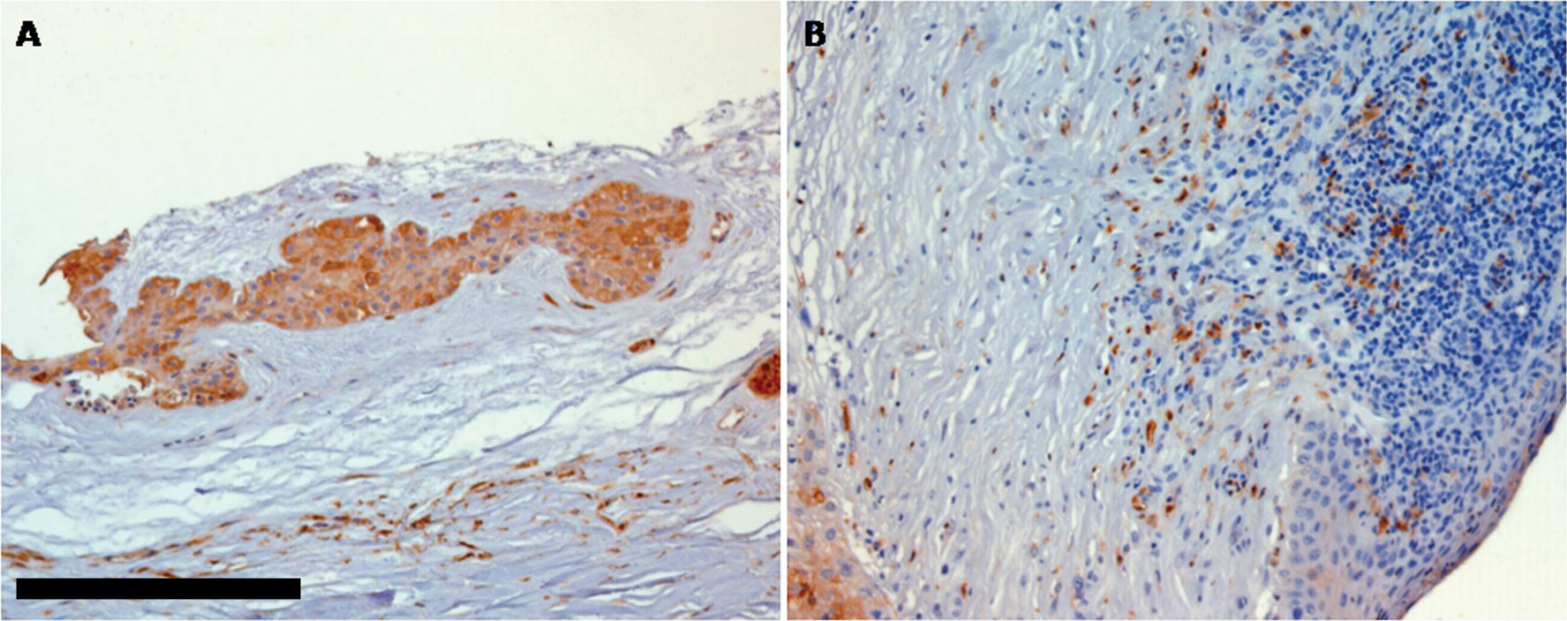
Immunohistochemical staining of total S100 in human healthy and inflamed limbal stem cell niche: The expression of S100 was localized in the cytoplasm of limbal cell niche (**A**) and was almost absent or faintly present in inflamed tissue (**B**). Each section was counterstained with hematoxylin. Bar scale=50 µm. Original magnification=200X.

Expression of S100 A and B was evaluated in healthy (nine samples) and inflamed (five samples) human limbus (Table 1). No staining was observed in a specific isotype control (results not shown). Strong cytoplasmic expression was observed in all LECs of healthy corneas (Figure 1A), while negative (4/5 samples, 80%) or a few positive cells were observed in inflamed limbus (1/5 samples 20%; Figure 1B).

**Table 1:** Total S100 expression in different human healthy and inflamed limbus samples.

Tissues	S100 positivity
Healthy limbus 1	++
Healthy limbus 2	++
Healthy limbus 3	++
Healthy limbus 4	++
Healthy limbus 5	++
Healthy limbus 6	++
Healthy limbus 7	++
Healthy limbus 8	++
Healthy limbus 9	++
Inflamed limbus 1	-
Inflamed limbus 2	-
Inflamed limbus 3	-/+
Inflamed limbus 4	-
Inflamed limbus 5	-

++: strong posititvity -/+: few positive cells -: no positive cells

### Expression of S100 protein members in normal human limbus

**Figure 2:**
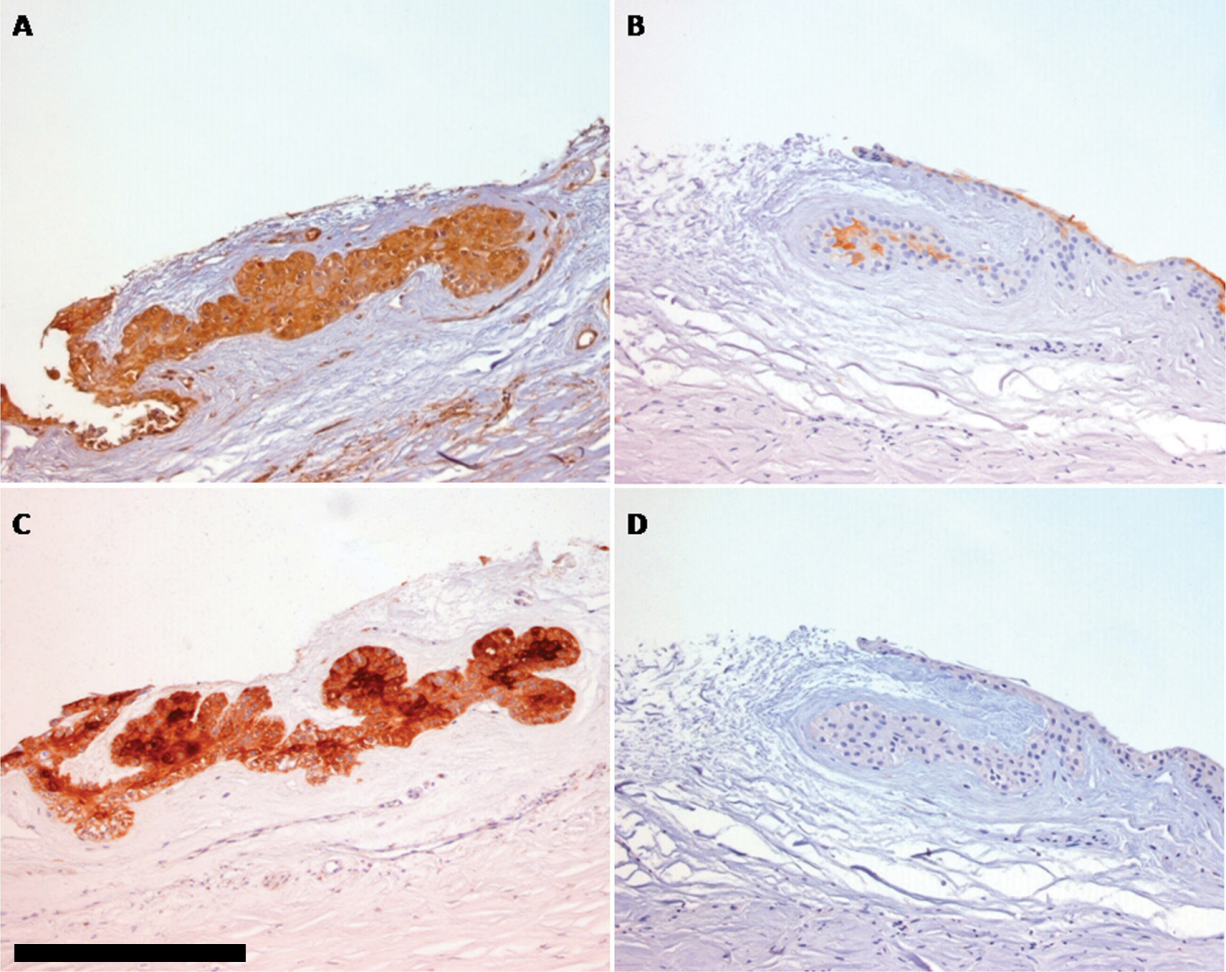
Expression of S100 A4, A8, A9, and B in human healthy limbal stem cell niche. S100 A4 and A9 positivity was evident in the entire limbal stem cell niche (**A**–**C**). In contrast, S100 A8 positivity was present in only a few cells in the middle area of the crypt (**B**). No positivity was achieved for S100 B staining (**D**). Each section was counterstained with hematoxylin. Bar scale=50 µm. Original magnification=200X.

Based on the strong S100 expression observed in healthy LECs, the individual localization of S100 A4, S100 A8, S100 A9, and S100 B proteins was analyzed. Strong S100 A4 staining was consistently observed in all samples (9/9 samples, 100%; Table 2). Cytoplasmic S100 A4 positivity was observed in the entire LEC (Figure 2A). Sporadic cytoplasmic positivity for S100 A8 antigen was observed in the center of the LEC (Figure 2B). Limbal crypts with S100 A8 positive cells were observed in 44.4% (4/9 samples) of analyzed cases (Table 2). Similarly to S100 A4, strong positivity for S100 A9 was found in all samples (Table 2 and Figure 2C). The expression of S100 B was not observed (Table 2 and Figure 2D).

**Table 2:** S100 A4, A8, A9, and B positivity in different samples of human healthy limbus.

Tissues	S100 positivity	S100 A4 positivity	S100 A8 positivity	S100 A9 positivity	S100 B positivity
Healthy limbus 1	++	+	+/−	++	-
Healthy limbus 2	++	+	-	++	-
Healthy limbus 3	++	++	-	++	-
Healthy limbus 4	++	++	-	++	-
Healthy limbus 5	++	++	-	++	-
Healthy limbus 6	++	++	+/−	++	-
Healthy limbus 7	++	++	-	++	-
Healthy limbus 8	++	++	+/−	++	-
Healthy limbus 9	++	++	+/−	++	-

++: strong posititvity -/+: few positive cells -: no positive cells

## Discussion

This study reports the expression of the S100 family proteins and the individual expression of S100 A4, A8, A9, and B members in normal human limbus (in particular in the LECs) and in eyes with inflamed limbus. LECs were first identified by Tseng [15] and then described by Dua et al. [17].

Recent studies have linked S100 positivity in the ocular surface tissue in proliferative, pathological inflammatory and non-inflammatory conditions [5]. Furthermore, a recent study by Jing and colleagues demonstrated differential expression and cellular distribution of multiple S100 A genes and proteins in normal corneal-limbal cells, suggesting that selective S100 proteins were involved in corneal epithelial cell proliferation and differentiation under normal and pathological conditions [16]. In our study, we tested four S100 members and in particular the S100 members that had been reported in the literature as playing a role in cellular growth and differentiation.

Recent studies linked S100 A4 to several diseases in addition to cancer, including kidney fibrosis [18-21], cirrhosis, pulmonary disease [22-24], cardiac hypertrophy and fibrosis [25-27], arthritis [28-31], and neuronal injuries [32-34]. The involvement of fibrotic and inflammatory processes was common to all these diseases, that is, processes greatly dependent on tissue remodeling, cell motility, and epithelial–mesenchymal transition.

Hence, S100 A4 interacts with cytoskeletal proteins and enhances metastasis of several types of cancer cells. In addition, S100 A4 is secreted by unknown mechanisms. This paracrine secretion stimulates various cellular responses, including angiogenesis and neuronal growth [35].

S100 A8 and A9 are mainly expressed in granulocytes and epithelial cells [16]. Secreted S100 A8 and S100 A9 proteins from the heterodimer are important members of damage-associated molecular proteins [36]. A study of a murine model of corneal neovascularization showed that S100 A8 and S100 A9 were involved in the inflammatory process; in particular, they facilitated the growth of new vessels [37].

S100 B is expressed in astrocytes and oligodendrocytes. Its concentration in cerebrospinal fluid (CSF) or serum is considered a suitable surrogate marker for the diagnostic and prognostic assessment of neurodegeneration (for example, in Alzheimer disease and amyotrophic lateral sclerosis). S100 B is not only implicated in regulating intracellular processes but is also a secretory protein exhibiting cytokine-like activities, which mediate interactions among glial cells and between glial cells and neurons [38]. To the best of our knowledge, this study provides the first illustration of limbal modifications induced by inflammation.

Since a history of corneal disease, such as documented keratitis, almost always precedes a diagnosis of endophthalmitis of different etiology [39], we speculate that a condition of ocular surface inflammation affecting sclerocorneal limbus is associated with flogistic processes that involve the entire ocular bulb. Moreover, in our pathological cases, all patients who underwent bulbar evisceration presented a specific history of ocular and or systemic diseases, associated with clinical signs of chronic ocular surface inflammation involving sclerocorneal limbus as documented by the inflammatory infiltration shown with immunohistochemistry analysis. Our results are certainly influenced by the differences in the pathophysiology of infection and limbal inflammation among the five cases analyzed. Moreover, patients who develop endophthalmitis are always in treatment with topical and or systemic steroids and antibiotics and often affected by systemic conditions associated with relative immune dysfunction [39-41]. These differences, which also concern our pathological cases, of course affect our results, by the way the minimal percentage of patients with endophthalmitis who undergo evisceration [39] make collecting samples difficult, and obtaining an homogeneous group for analysis is hard.

In our study, we observed strong cytoplasmic expression of the total S100 family in all LECs of healthy corneas, while only a few positive cells were observed in inflamed limbus. This may involve different considerations. First, the drastic reduction in S100 expression, highlighted in limbal inflammation, showed a different behavior of this family of proteins in limbal tissues compared to the other forms of ocular surface inflammation in which S100 overexpression was reported [6-14]. Furthermore, this consideration supports the hypothesis that S100 proteins represent a marker of limbal normality. Moreover, A4 and A9 presented higher limbal expression in our healthy samples (Figure 2).

These considerations confirm the results already reported in literature, indicating the role of S100 A4 and A9 in cell differentiation, regulation of growth, and cellular structure, processes observed in the sclerocorneal limbus [15]. In particular, S100 A4 overexpression in tumor cells suggests that this protein plays a role in the expression of the transformed phenotype [1] similarly to the processes that control corneal epithelial stem cells at the limbus LECs [15,38].

Poor S100 A8 expression and the absence of S100 B (Figure 2) could be explained by the fact that these proteins are probably involved in processes that are not primarily found at the limbus (for example, response to chemotactic factors and regulation kinase activity [1] for A4 or cellular learning and memory processes for S100 B [42]). Further examinations and an increase in the number of healthy and pathological samples would help confirm these findings and lead to better understanding of the function of S100 proteins and their role at the limbus. These preliminary results further elucidate how the sclerocorneal limbus holds features that make it completely different from the other elements that constitute the ocular surface, and a complex structure that has yet to be investigated and discovered.
